# Postpartum Eosinophilic Ascites: A Case Report

**DOI:** 10.7759/cureus.23301

**Published:** 2022-03-18

**Authors:** Inês Gonçalves, Ana Filipa S Pinho, Ana Antunes, Sofia Carvalho, Luisa Pinto

**Affiliations:** 1 Department of Internal Medicine, Hospital de Braga, Braga, PRT; 2 Department of Intensive Care, Hospital de Braga, Braga, PRT; 3 Department of Emergency Medicine, Hospital de Braga, Braga, PRT; 4 Department of Pathology, Hospital de Braga, Braga, PRT

**Keywords:** hypereosinophilia, eosinophilic gastroenteritis, ascites, postpartum, hypereosinophilic syndrome

## Abstract

Eosinophilic gastrointestinal diseases (EGID) are a group of conditions characterized by histopathologic eosinophilic infiltrates in one or more segments of the gastrointestinal (GI) tract. It occurs in the absence of known causes for eosinophilia. It can affect every part of the gastrointestinal tract, but eosinophilic ascites (EA) is uncommon. There is a clinical overlap between EGID and GI involvement of hypereosinophilic syndrome (HES), so distinguishing them may not be easy.

We report a case of eosinophilic gastroenteritis in a 26-year-old-woman with the uncommon presentation of eosinophilic ascites after delivery. It is vital to maintain a high grade of suspicion to diagnose these disorders and exclude the secondary causes since treatment varies. In addition, the occurrence of this postpartum syndrome has been described, so it is essential to recognize this entity in this period.

## Introduction

Eosinophilic gastrointestinal diseases (EGID) are a group of diseases characterized by histopathologic eosinophilic infiltrates in one or more segments of the gastrointestinal tract. It occurs in the absence of known causes for eosinophilia (parasitic or fungal infections, allergic, immunological disorders, or medication-induced) [1].

Although rare, its actual prevalence is unknown. It can affect every age group but has a peak of onset in the third decade. Even though is found mainly in males, eosinophilic ascites is more common in women [1,2]. Its pathogenic mechanisms are not fully understood, but multiple epidemiologic and clinical features suggest an allergic component, with a large proportion of patients (70%) presenting with concomitant atopic conditions [1,3].

EGID symptoms may vary depending on the location of the gastrointestinal tract affected and the gut layer involved: mucosal, muscular, and subserosal. The mucosal form produces non-specific symptoms like abdominal pain, nausea, vomiting, diarrhea, and, less commonly, weight loss and malnutrition due to protein-losing enteropathy. Muscular involvement results in wall thickening and impaired motility and may present with intestinal obstruction or perforation. The serosal form is very rare and leads to eosinophilic ascites [2].

## Case presentation

A previously healthy 26-year-old woman presented to the emergency department with complaints of intermittent abdominal pain and distention, nausea, nonbilious and non-bloody vomiting for three weeks, and weight gain of 7 kg. The abdominal pain was dull aching, and diffuse, lasting for several minutes and resolving spontaneously. She denied any associated symptoms. The patient had an uneventful pregnancy and delivered 10 weeks earlier a healthy infant without complications. She had already been observed by a gynecologist who excluded obstetric complications and was discharged on proton pump inhibitors without clinic response. 

She was not taking any chronic medication or supplements and denied consuming alcohol or illicit drugs, recent travel, transfusions, allergies, or ill contact. Familiar history was unremarkable. She was alert, afebrile, and hemodynamically stable. Her abdomen was distended and tender diffusely, with shifting dullness. There were no caput medusae, rebound, or guarding. No hepatosplenomegaly was found. The rest of her physical examination was normal. Blood analyses were obtained, revealing leukocytosis with significant eosinophilia and no immature myeloid precursors, without other findings (Table 1). 

**Table 1:** Blood and peritoneal fluid analytics from emergency department on initial approach.

Blood analyses	Result	Reference values
Hemoglobin	15.1 g/dL	11.9-15.6
Hematocrit	43.8%	36.6-45.0
Mean corpuscular volume	91.3 fl	82.9-98.0
Mean corpuscular hemoglobin concentration	32.5 pg	27.0-32.3
Leucocytes	19.1 × 10^3^/uL	4.0-11.0
Neutrophils	7.6 × 10^3^/uL (40%)	1.8-7.1
Eosinophils	8.4 × 10^3^/uL (44%)	0.0-0.5
Lymphocytes	2.5 × 10^3^/uL (13%)	1.2-3.4
Monocytes	0.6 × 10^3^/uL (3%)	0.2-0.9
Urea	21 mg/dL	15-39
Creatinine	0.9 mg/dL	0.6-1.20
Potassium	4.3 mmol/L	3.5-5.1
Sodium	143 mmol/L	136-145
Total bilirubin	0.57 mg/dL	0.1-1.9
Conjugated bilirubin	0.15 mg/dL	0-0.2
Aspartate aminotransferase test	8 U/L	15-37
Alanine aminotransferase test	17 U/L	12-78
Amylase	31 U/L	25-115
Lipase	101 U/L	73-393
Alkaline phosphatase	68 U/L	45-117
Lactate dehydrogenase	211 U/L	84-246
Total creatinine kinase	27 U/L	26-192
Troponin I	< 0.02 ng/mL	<0.045
Myoglobin	23 ng/mL	13-71
N-terminal pro-B-type natriuretic peptide	94.0 pg/mL	<125
C-reactive protein	11.10 mg/L	<3.0
Total protein	6.7 g/dL	6.4-8.2
Albumin	3.5 g/dL	3.4-5.0
Thyroid stimulating hormone	0.967 UI/mL	0.358-3.74
Erythrocyte sedimentation rate	2 mm/h	1-20
Peritoneal fluid analyses		
Glucose	83 mg/dL	-
Total proteins	4.7 g/dL	-
Lactate dehydrogenase	169 UI/L	-
Albumin	2.7 g/dL	-
Erythrocytes	15.100/uL	-
Leukocytes	10.585/uL	-
Eosinophils	85%	-
Lymphocytes	3%	-
Neutrophils	2%	-
Monocytes	6%	-
Macrophages	2%	-
Citology for neoplastic cells	Negative	-

On abdominal and pelvic ultrasonography, the liver had normal size and echogenicity, but a large amount of peritoneal fluid was identified. Diagnostic paracentesis was performed, obtaining a hazy dark yellow, with low serum-ascites albumin gradient and significant eosinophilia (85%). Mycobacterial and microbiological cultures were negative. No cytological signs of malignancy were found. The diagnostic approach facing these findings included an etiologic study and an evaluation of end-organ damage. We found peripheral smear, serum immunoglobulin E, metabolic, immunological panels, and parasitic studies to be normal or negative (Table 2).

**Table 2:** Blood analytics from hospital internment for etiologic study.

Blood analytics	Result	Reference value
Human immunodeficiency virus type 1 and 2 serology	Non-reactive	-
Hepatitis B surface antigen	Non-reactive	-
Hepatitis B surface antibody	Positive	-
Total hepatitis B core antibody	Non-reactive	-
Cytomegalovirus serology	Non-reactive; no previous contact	-
Venereal disease research laboratory test	Non-reactive	-
Epstein‐Barr virus serology	Previous contact; no active infection	-
Anti-herpes virus type 1	Previous contact; no active infection	-
Anti-herpes virus type 2	Non-reactive	-
Fecal examination	Negative for parasites (3 samples)	-
Serum protein electrophoresis	Albumin: 3.1 g/dL	3.4-5
	Alfa 1: 0.4 g/dL	0.2-0.4
	Alfa 2: 0.4 g/dL	0.5-1
	Beta 1: 0.3 g/dL	0.3-0.6
	Beta 2: 0.3 g/dL	0.2-0.5
	Gamma: 0.7 g/dL	0.7-1.6
Immunoelectrophoresis	No monoclonal peaks were found	-
Immunoglobulin A	72 mg/dL	70-400
Immunoglobulin M	41.2 mg/dL	40-230
Total immunoglobulin E	41.4 UI/mL	<129
Immunoglobulin G	703 mg/dL	700-1600
Rheumatoid factor	<10 UI/mL	<15
Complement component 3	114 mg/dL	90-180
Complement component 4	50 mg/dL	14-45
Antinuclear antibodies	Non-reactive	-
Perinuclear anti-neutrophil cytoplasmic antibodies	3.1 U/mL	<5
Proteinase 3 anti-neutrophil cytoplasmic antibodies	2 U/mL	<5
Interferon-gamma release assay	Negative	-

**Figure 1:**
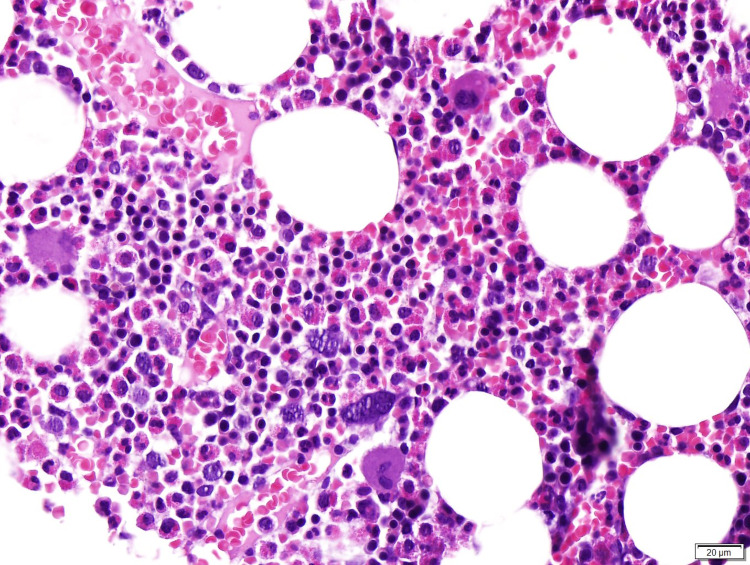
Photomicrography of bone marrow (hematoxylin and eosin, 400x). The image illustrates no lymphoid infiltrates, adequate trilineage maturation, no blast, and 20% eosinophils.

**Figure 2:**
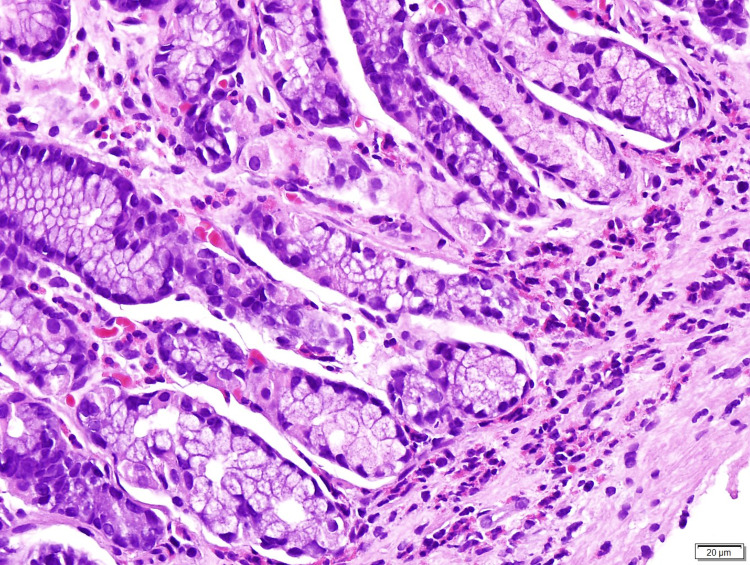
Photomicrography of a gastric mucosal biopsy specimen (hematoxylin and eosin, 400x). The image is showing preserved glandular component with more than 30 eosinophils per high power field, with chronic superficial gastritis.

A bone marrow aspirate was performed, showing no lymphoid infiltrates, adequate trilineage maturation, no blast, and 20% eosinophils, without cytogenetic abnormalities identified (Figure 1). Esophagogastroduodenoscopy revealed mild gastric mucosal erythema. Gastric biopsies showed preserved glandular component with eosinophils infiltration, with chronic superficial gastritis, negative for dysplasia and *Helicobacter pylori* (Figure 2). 

**Figure 3:**
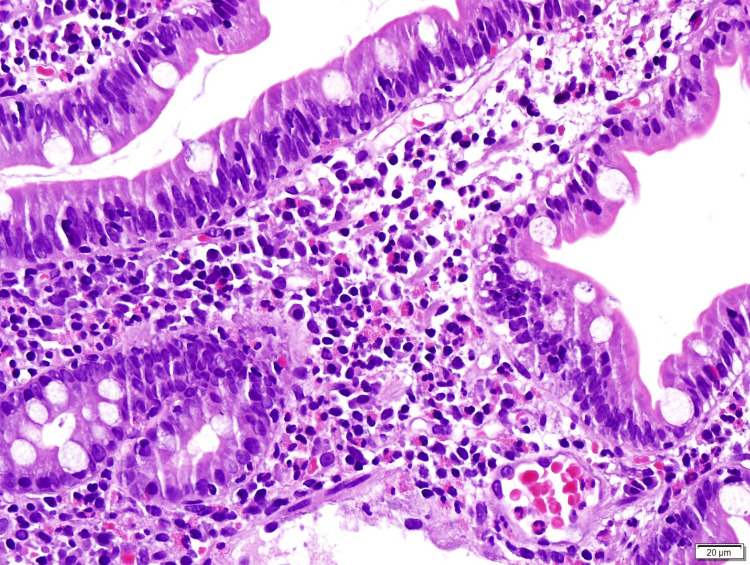
Photomicrography of a duodenal mucosal biopsy specimen (hematoxylin and eosin, 400x). The image is showing preserved glandular component, with more than 50 eosinophils per high power field in the intraepithelial mucosa, extending into the muscularis and serosa.

Duodenal biopsies revealed more than 50 eosinophils per high power field in the intraepithelial mucosa, extending into the muscularis and serosa (Figure 3). Colonoscopy was also realized, with biopsies showing normal histology. In addition, we ordered troponin T, electrocardiogram, echocardiogram, pulmonary function tests, thoracic computed tomography, and urinalysis to exclude end-organ damage. All of them without remarkable findings. 

A diagnosis of eosinophilic gastrointestinal disease with mucosal, muscular, and serosal involvement was then established. Since we observed eosinophilic infiltration in gastric and duodenal biopsies and eosinophilic ascites, we decide not to pursue diagnostic laparoscopy and peritoneal biopsy. Our patient was started on 0.5 mg/kg of daily oral prednisone. After two weeks, her symptoms resolved entirely, and a gradual steroid taper was initiated. The patient was followed-up monthly with a complete blood count. Her eosinophil counts gradually decreased and returned to normal in four months. A complete steroid taper was achieved, and after a follow-up period of one year, she remains asymptomatic, and her eosinophil counts are within normal limits.

## Discussion

In patients with EGID, peripheral eosinophil counts are usually elevated, but they can be normal in 20% of patients. Currently, hypereosinophilia is defined as a persistence of peripheral blood eosinophilia over 1500 cells/μL on at least two occasions, four weeks apart, or evidence of marked tissue eosinophilia. When accompanied with clear evidence of more than one organ damage, it would be called hypereosinophilic syndrome (HES) [4]. Nevertheless, eosinophilic gastrointestinal disease is excluded and classified as organ-restricted hypereosinophilic conditions [2]. These disorders can be challenging to distinguish from idiopathic HES, and lack of involvement of extraintestinal organs must be confirmed.

In our case, the presence of eosinophilic infiltration on biopsies from stomach and duodenum (greater than or equal to 30 eosinophils per high-power field) and the absence of extraintestinal disease support EGID diagnosis [5]. Although laparoscopic biopsies may be required for a definitive diagnosis of serosal involvement, we considered the three-layer involvement for the following reasons: the existence of eosinophilic ascites; the presence of eosinophilic infiltrate extending to the muscularis and serosal on duodenal biopsy; and, finally, the dramatic response to steroid therapy.

Regardless, it is vital to notice that disease can progress to extraintestinal involvement in patients with EGID and hypereosinophilia [3]. Furthermore, patients should be referred for appropriate hematological evaluation when EGID presents as a part of HES because eventual malignant transformation is possible [2].

There is no consensus about EGID treatment, but the main goal is to reduce eosinophil load. The treatment with steroids shows improvement in most cases. Steroid strategies include topical glucocorticoids, such as enteric coat budesonide, or more commonly, oral prednisolone (typically 0.5 a 1 mg/kg/day) [1]. In non-responders or for corticosteroid sparing purposes, novel approaches are being used, including leukotriene receptor antagonists and monoclonal antibodies against IgE and IL-5. However, further in-depth studies are necessary [2].

Our case has some unique features. On the one hand, EGID presenting with ascites is a rare condition. On the other hand, most reported cases of eosinophilic ascites are idiopathic, and they are often accompanied by an atopic condition such as asthma or a food or medication allergy. In addition, it is very unusual for EGID or eosinophilic ascites (EA) to be precipitated by pregnancy or childbirth. Only a few other cases of EA postpartum have been reported, and recurrence of the symptoms with succeeding pregnancies have been described [2].

## Conclusions

Eosinophilic gastrointestinal diseases are a group of heterogeneous diseases characterized by eosinophilic infiltration of the gastrointestinal tract, and they can present as a variety of gastrointestinal symptoms. High suspicion is necessary for the diagnosis of these disorders, especially if systemic eosinophilia is absent. Despite being a rare cause, EGID should be considered in the study of ascites in the absence of liver disease. Secondary causes must be excluded in the presence of gastrointestinal eosinophilia. This case highlights the occurrence of this syndrome during the postpartum period and enhances the importance of including EGID in the differential diagnoses of postpartum gastrointestinal disorders.
